# A Computer Application to Predict Adverse Events in the Short-Term Evolution of Patients With Exacerbation of Chronic Obstructive Pulmonary Disease

**DOI:** 10.2196/10773

**Published:** 2019-04-17

**Authors:** Inmaculada Arostegui, María José Legarreta, Irantzu Barrio, Cristobal Esteban, Susana Garcia-Gutierrez, Urko Aguirre, José María Quintana, IRYSS-COPD Group

**Affiliations:** 1 Departamento de Matemática Aplicada y Estadística e Investigación Operativa The University of the Basque Country UPV/EHU Leioa Spain; 2 Research Institute Basque Center for Applied Mathematics Bilbao Spain; 3 Red de Investigación en Servicios Sanitarios en Enfermedades Crónicas Galdakao Spain; 4 Unidad de Epidemiología Clínica Hospital Galdakao Galdakao Spain; 5 Servicio de Neumología Hospital Galdakao Galdakao Spain

**Keywords:** COPD, disease exacerbation, mortality, intensive care, clinical prediction rule, mobile app

## Abstract

**Background:**

Chronic obstructive pulmonary disease (COPD) is a common chronic disease. Exacerbations of COPD (eCOPD) contribute to the worsening of the disease and the patient’s evolution. There are some clinical prediction rules that may help to stratify patients with eCOPD by their risk of poor evolution or adverse events. The translation of these clinical prediction rules into computer applications would allow their implementation in clinical practice.

**Objective:**

The goal of this study was to create a computer application to predict various outcomes related to adverse events of short-term evolution in eCOPD patients attending an emergency department (ED) based on valid and reliable clinical prediction rules.

**Methods:**

A computer application, Prediction of Evolution of patients with eCOPD (PrEveCOPD), was created to predict 2 outcomes related to adverse events: (1) mortality during hospital admission or within a week after an ED visit and (2) admission to an intensive care unit (ICU) or an intermediate respiratory care unit (IRCU) during the eCOPD episode. The algorithms included in the computer tool were based on clinical prediction rules previously developed and validated within the Investigación en Resultados y Servicios de Salud COPD study. The app was developed for Windows and Android systems, using Visual Studio 2008 and Eclipse, respectively.

**Results:**

The PrEveCOPD computer application implements the prediction models previously developed and validated for 2 relevant adverse events in the short-term evolution of patients with eCOPD. The application runs under Windows and Android systems and it can be used locally or remotely as a Web application. Full description of the clinical prediction rules as well as the original references is included on the screen. Input of the predictive variables is controlled for out-of-range and missing values. Language can be switched between English and Spanish. The application is available for downloading and installing on a computer, as a mobile app, or to be used remotely via internet.

**Conclusions:**

The PrEveCOPD app shows how clinical prediction rules can be summarized into simple and easy to use tools, which allow for the estimation of the risk of short-term mortality and ICU or IRCU admission for patients with eCOPD. The app can be used on any computer device, including mobile phones or tablets, and it can guide the clinicians to a valid stratification of patients attending the ED with eCOPD.

**Trial Registration:**

ClinicalTrials.gov NCT00102401; https://clinicaltrials.gov/ct2/show/results/NCT02434536 (Archived by WebCite at http://www.webcitation.org/76iwTxYuA)

**International Registered Report Identifier (IRRID):**

RR2-10.1186/1472-6963-11-322

## Introduction

Chronic obstructive pulmonary disease (COPD) is one of the most common chronic diseases, and its prevalence is expected to increase over the next few decades [1]. COPD is a leading cause of death in developed countries, and patients with COPD generally suffer a substantial deterioration in their quality of life [2]. COPD is a complex and heterogeneous condition with different clinical manifestations and variable disease activity. There is a continuing interest in using clinical and pulmonary function variables and other disease indicators that may help predict outcomes [3].

The exacerbation of COPD (eCOPD) is defined as an event in the natural course of a patient’s COPD characterized by a change in baseline dyspnea, cough, or sputum, that is beyond normal day-to-day variations and that may have warranted a change in medication or treatment [4]. Exacerbations are common among patients with COPD [5]. These sudden worsenings of COPD contribute to disease progression, reduce quality of life, increase the risk of death, and account for substantial use of health care resources [2,6,7]. Currently, emergency department (ED) physicians must rely largely on their experience and the patient’s personal criteria to gauge how an eCOPD will evolve. Clinical prediction rules that could help predict eCOPD evolution would allow ED physicians to make better-informed decisions about treatment [8].

Prediction models are gaining importance as a support for decision-making processes. Decisions such as the most appropriate treatment for a disease; whether or not a given patient should be discharged; or the development of effective, acceptable, and cost-efficient prevention strategies are based on the individual patient’s risk of suffering some undesirable event. Clinical prediction models provide estimates for an individual’s risk of an adverse event over a specific period on the basis of a combination of a number of patient characteristics, which we call variables. Often, clinical prediction models are extended to include clinical prediction rules, risk scores, or prognostic models. The literature includes well-known prediction models, which have been developed to predict the development of a disease, death, or poor evolution caused by a current disease, including eCOPD. More precisely, the Investigación en Resultados y Servicios de Salud COPD (IRYSS-COPD) Appropriateness Study group has developed clinical prediction rules for short-term outcomes in eCOPD patients attending an ED. These outcomes include (1) mortality during hospital admission or within a week after the ED visit [9] and (2) admission to an intensive care unit (ICU) or an intermediate respiratory care unit (IRCU) during the eCOPD episode [10].

Nowadays, clinicians and patients are both actively involved in deciding therapeutic interventions or choosing medical treatments in a shared decision-making process. It is well known that the estimation of an individual’s risks of various adverse events by means of prediction models may provide the necessary input for shared decision-making [11]. Therefore, the application of clinical prediction rules in daily clinical practice is one more step in this process. The translation of clinical prediction rules into easy-to-use computer tools would allow the use of these models in clinical practice. The goal of this work was to create a computer application to predict various outcomes related to adverse events of short-term evolution in eCOPD patients attending an ED based on valid and reliable clinical prediction rules. We present the Prediction of Evolution of patients with eCOPD (PrEveCOPD) tool for prediction of 2 outcomes: (1) mortality during hospital admission or within a week after the ED visit and (2) admission to an ICU or IRCU during the eCOPD episode. The algorithms included in the computer tool are based on the clinical prediction rules previously published by Quintana et al [9,10] for the IRYSS-COPD study.

The rest of the paper is organized as follows. The Methods section presents a brief description of the IRYSS-COPD study, including the development of the predictive models and clinical rules, and provides the methodology used to create the PrEveCOPD computer tool for different environments. The Results section describes the PrEveCOPD tool and shows how it runs with individual cases. Finally, the paper closes with a discussion in which the novelty and usefulness of the application, some limitations, and future work are reviewed and conclusions are drawn.

## Methods

### The Investigación en Resultados y Servicios de Salud-Chronic Obstructive Pulmonary Disease Study: Description and Outcome Prediction Rules

A detailed description of the IRYSS-COPD study has been reported in depth in the study protocol [12]. In brief, this prospective cohort study included subjects with an eCOPD attending the ED of 16 hospitals in Spain between June 2008 and September 2010. The study was approved by the institutional review boards of the participating hospitals, in accordance with all applicable regulations. All patients were informed of the goals of the study and invited to voluntarily participate in it; confidentiality was guaranteed. All who agreed to participate provided written consent.

Data from several time points were collected in the study. However, for the purpose of this study, we concentrated on variables collected at 2 time points. First, data were collected when the decision was made to hospitalize the patient or discharge him or her home. If the patient was hospitalized, then additional data were collected in the medical ward up to 1 week. Otherwise, if the patient was discharged, he or she was contacted by phone, and similar information was recorded up to 1 week after the index ED visit. The selected predictive variables were previously described [12]. The selected 2 outcome variables were also previously described when the predictive models were developed [9,10]. However, because of the importance of the 2 outcomes for the purpose of this study, we present a brief definition of them. The 2 outcome variables were as follows:

- Death, if it occurred during the hospital admission or within 7 days of the index ED visit among patients discharged to home.
- ICU or IRCU admission: The patient needs an ICU admission or invasive mechanical ventilation (IMV) or suffers a cardiac arrest; or the patient needs a noninvasive mechanical ventilation (NIMV) for 2 or more days, when mechanical ventilation was not used at home before admission or needs an admission to an IRCU for 2 or more days. A minimum of 2 days was chosen to include only those patients needing more intensive and prolonged therapeutic interventions.

This description is restricted to the variables finally considered for the development of the 2 prediction rules. Table 1 shows the distribution of the selected predictive variables by outcome.

The 2 clinical prediction rules were developed following similar methodological approaches. Detailed description is provided elsewhere [9,10], although a brief summary is given below.

Univariate logistic regression analysis was initially performed, and variables with statistically significant results at *P*<.20 were posteriorly entered into a multiple logistic regression model. Internal validation of the variable selection process and modeling was performed until the final predictive model was reached. A score was developed by assigning a weight to each variable or category in the final multiple logistic regression model, as suggested in the literature [13]. Finally, the score was categorized into a manageable number of risk classes based mainly on the estimated risk of event for each outcome.

**Figure 1:**
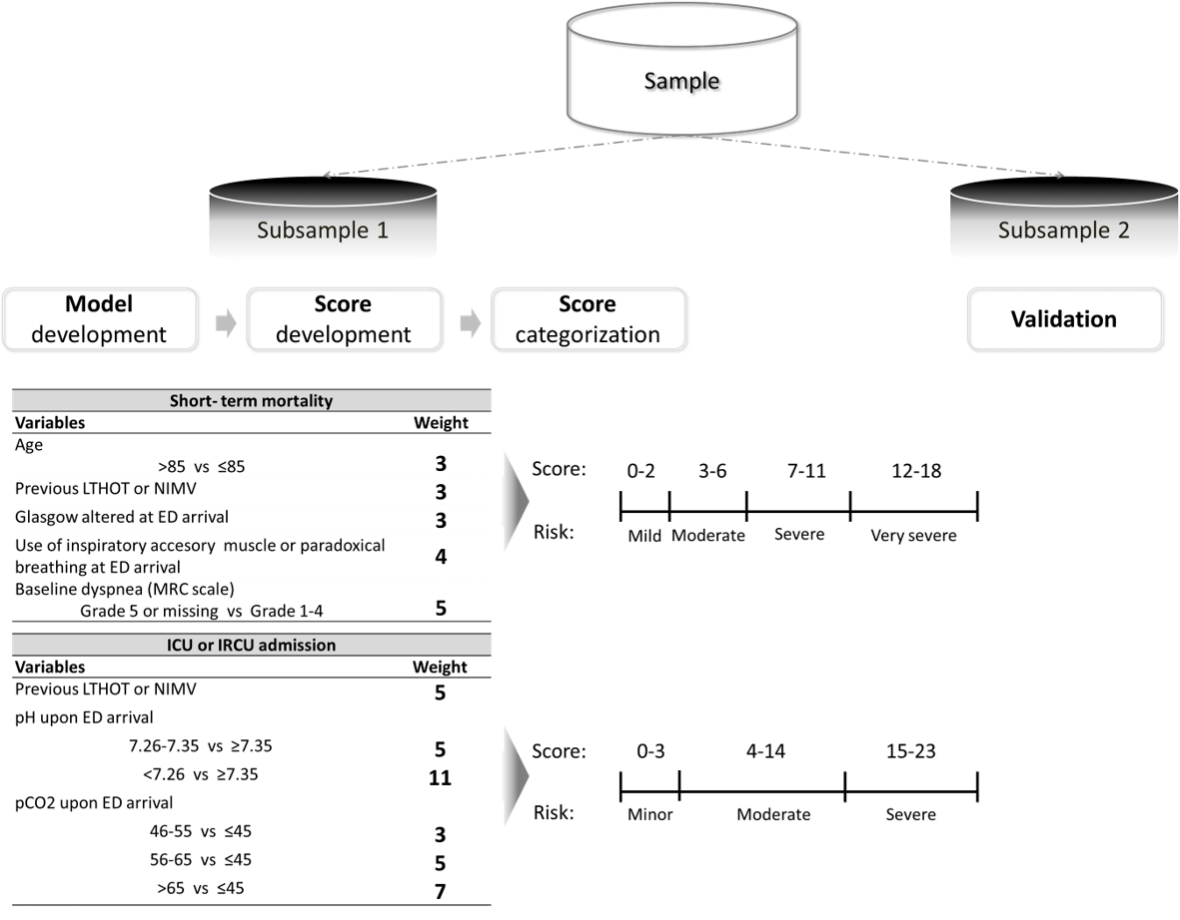
Summary of the process for the 2 outcomes (death and intensive care unit or intermediate respiratory care unit admission): score development and stratification into risk categories. ED: emergency department; ICU: intensive care unit; IRCU: intermediate respiratory care unit; LTHOT: long-term home oxygen therapy; MRC: Medical Research Council; NIMV: noninvasive mechanical ventilation; P_CO2_: pressure of carbon dioxide.

Discrimination of the score and the risk categories was assessed by the area under the receiver operating characteristic curve. All the modeling, scoring, and categorization processes were validated by split-sample validation (50% development and 50% validation). Figure 1 shows the whole process of score development and categorization and the resulting risk categories for the 2 outcomes, death and ICU or IRCU admission. The Cochran-Armitage trending statistic was performed to assess whether classification provided by the score could differentiate low-risk patients from high-risk patients in a fashion of graded response based on the level of risk present.

Results of the developed risk categories and association with the 2 outcomes are shown in Table 2. Note that because of missing values in predictor or response variables, the total number of subjects for which the 2 risk scores were estimated differed. Detailed information regarding missing values can be obtained in the original papers where these scores were developed.

### The Computer Application: Prediction of Evolution of Patients With Exacerbation of Chronic Obstructive Pulmonary Disease

The PrEveCOPD computer application has been implemented to be installed both in Windows and Android systems and can also be used on the Web without installing any application.

The application for Windows and Web platforms has been developed using Microsoft Visual Studio 2008 [14], and a tool called Eclipse [15] was used to develop the instrument to be run on an Android system.

For the Windows application, we used C# programming language [16] to develop the application and then install and run locally on the user’s computer. The Web application was also created in C# and implemented on a computer workstation so that users could access it remotely. The application is available for downloading and installing on the computer or to be used remotely as a Web application [17]. Therefore, anyone with an internet connection and browser could access the website and run the application. The application operates exactly in the same way when the access is local and remote. The performance of the Windows application has been checked under Windows 7, in a 32-bits personal computer. The most common browsers (Internet Explorer, Firefox, Chrome, and Safari), with updated plugins installed for Java version 8 or posterior, have been tested for the Web application. For the Android app, we used the Java programming language to develop the app in the Eclipse development environment and install it and run it on any device with an Android operating system. The Android app is available on Google Play under the medicine category, with the name PrEveCOPD. The performance of the app has been tested under Android version 7.0 or posterior.

The minimum equipment requirements that we recommend to run the application are Windows 7 with 32 bits and 4 GB of RAM memory for local access under Windows, Java version 8, and one of the following browsers: Internet Explorer 11, Firefox 59, or Chrome 69 for remote access or Android Nougat 7.0 release for the Android app.

**Figure 2:**
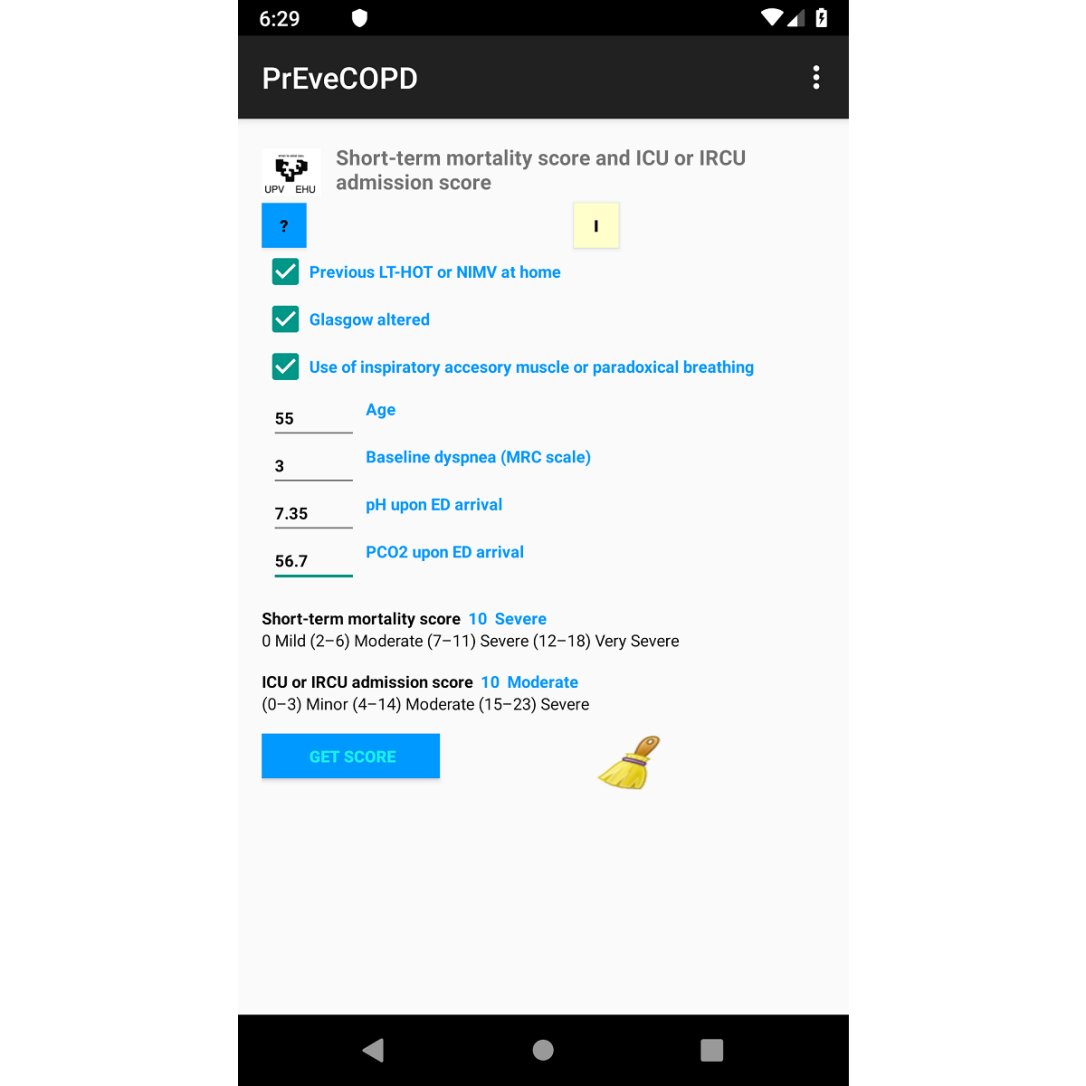
Screenshot of the application running under the Android platform. Data for an imaginary subject with complete information displayed as an example. ED: emergency department; ICU: intensive care unit; IRCU: intermediate respiratory care unit; LTHOT: long-term home oxygen therapy; MRC: Medical Research Council; NIMV: noninvasive mechanical ventilation; P_CO2_: pressure of carbon dioxide.

**Figure 3:**
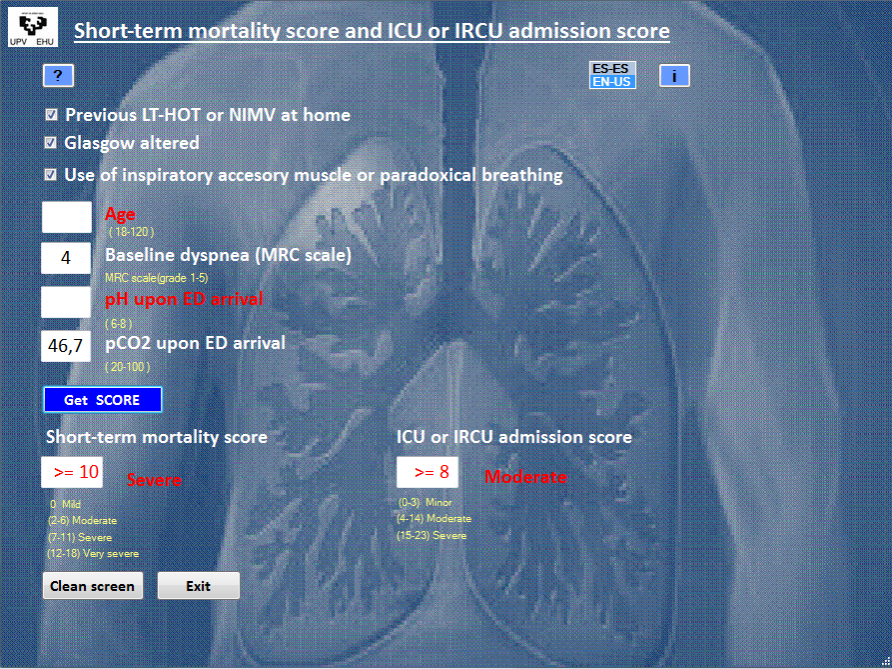
Screenshot of the application running under Windows and Web platforms. Data for an imaginary subject with incomplete information displayed as an example. ED: emergency department; ICU: intensive care unit; IRCU: intermediate respiratory care unit; LTHOT: long-term home oxygen therapy; MRC: Medical Research Council; NIMV: noninvasive mechanical ventilation; P_CO2_: pressure of carbon dioxide.

Figures 2 and 3 show a screenshot of the Android app running on a mobile phone (Figure 2) and the tool under Windows (Figure 3). The computer application has been developed in English and Spanish. For electronic devices running under Android, the language is automatically detected depending upon the default settings, with English being the default option for any language other than Spanish. For a computer running under Windows, the application has an option to switch between the 2 languages, with Spanish being the default option.

**Table 1:** Distribution of the predictive variables by outcome. The 2 outcomes are mortality during hospital admission or within a week after the emergency department visit and admission to an intensive care unit or intermediate respiratory care unit during the exacerbation of chronic obstructive pulmonary disease episode (N=2487).

Predictive variable			Sample, n (%)		Mortality					Admission to intensive care unit or intermediate respiratory care unit		
					n (%)		*P* value^a^		n (%)			*P* value^a^
Total					59 (2.37)		—^b^		258 (10.37)			—
**Age (years)**												
	<75	1271 (51.11)		18 (1.42)		<.001		166 (13.06)			<.001	
	75-85	1050 (42.22)		28 (2.67)		<.001		80 (7.62)			<.001	
	>85	165 (6.63)		13 (7.88)		<.001		12 (7.27)			<.001	
**Previous long-term home oxygen therapy or noninvasive mechanical ventilation**												
	Yes	841(33.82)		43 (5.11)		<.001		190 (22.59)			<.001	
	No	1646 (66.18)		16 (0.97)		<.001		68 (4.13)			<.001	
**Altered consciousness**												
	Yes	70 (2.81)		9 (12.86)		<.001		36 (51.43)			<.001	
	No	2415 (97.10)		49 (2.03)		<.001		220 (9.11)			<.001	
**Use of inspiratory accessory muscle**												
	Yes	535 (21.51)		34 (6.36)		<.001		111 (20.75)			<.001	
	No	1952 (78.49)		25 (1.28)		<.001		147 (7.53)			<.001	
**Dyspnea (Medical Research Council)**												
	Missing	250 (10.05)		18 (7.20)		<.001		21 (8.40)			<.001	
	Grade 1	188 (7.56)		0 (0)		<.001		10 (5.32)			<.001	
	Grade 2	600 (24.13)		1 (0.17)		<.001		44 (7.33)			<.001	
	Grade 3	501 (20.14)		7 (1.40)		<.001		45 (8.98)			<.001	
	Grade 4	672 (27.02)		10 (1.49)		<.001		81(12.05)			<.001	
	Grade 5	276 (11.10)		23 (8.33)		<.001		57 (20.65)			<.001	
**pH**												
	≥7.35	1991 (86.75)		40 (2.01)		.02		121 (6.08)			<.001	
	7.26-7.35	250 (10.89)		11 (4.40)		.02		87 (34.80)			<.001	
	<7.26	54 (2.35)		3 (5.56)		.02		38 (70.37)			<.001	
**Pressure of carbon dioxide (P_CO2_)**												
	≤45	1232 (57.20)		16 (1.30)		<.001		32 (2.60)			<.001	
	45-55	484 (22.47)		14 (2.89)		<.001		47 (9.71)			<.001	
	55-65	241 (11.19)		10 (4.15)		<.001		58 (24.07)			<.001	
	>65	197 (9.15)		13 (6.60)		<.001		107 (54.31)			<.001	

^a^Chi-square test for homogeneity.

^b^Not applicable.

The main screen incorporates a help button, where the specific definition of all the predictive variables is detailed exactly the same as in the manuscripts where prediction rules were developed [9,10]. The computer tool also incorporates a predefined range of acceptable values for each variable to control for typing mistakes or out-of-range values. An error message prevents invalid values to be introduced, with strict instructions about the accepted range of values. The application accepts a missing value in any of the predictive variables, leading in that case to a lower bound for the corresponding score.

A button with information for users about the legal responsibility derived from the use of the application is also incorporated in the Android platform, and this information is displayed on the main screen in the Windows and Web platforms.

**Table 2:** Distribution of the developed risk categories for each of the outcomes.

Outcome		Yes, n (%)	No, n (%)	*P* value^a^
**Short-term mortality risk**				
	Mild (n=1081)	3 (0.28)	1078 (99.72)	<.001
	Moderate (n=865)	11 (1.27)	854 (98.73)	<.001
	Severe (n=441)	20 (4.54)	421 (95.46)	<.001
	Very severe (n=97)	24 (24.74)	73 (75.26)	<.001
**Intensive care unit or intermediate respiratory care unit admission risk**				
	Minor (n=1203)	12 (1.00)	1191 (99.00)	<.001
	Moderate (n=803)	144 (17.93)	659 (82.07)	<.001
	Severe (n=148)	88 (59.46)	60 (40.54)	<.001

^a^Cochran-Armitage trend-test.

## Results

The final product is an application with a user-friendly interface that comprises a screen where the values of the specific predictive variables are introduced. Then, by pushing the *Get SCORE* button, the estimated score for the 2 outcomes, death and ICU or IRCU admission, are automatically shown. The screen also shows the stratification of risk into categories for both scores.

Furthermore, 5 parameters defined the final model for predicting death during hospital admission or within 1 week of discharge from the ED to home: age, previous history of long-term home oxygen therapy (LTHOT) or need for NIMV, altered consciousness measured by Glasgow coma scale (GCS), use of accessory inspiratory muscles or paradoxical breathing upon ED arrival, and baseline dyspnea measured by the Medical Research Council (MRC) scale. The final predictive model for ICU or IRCU admission was defined by 3 variables. One of them was the same as in the previous model for death, namely, previous history of LTHOT or need for NIMV. The other 2 were elevated P_CO2_ and decreased pH upon ED arrival. Previous history of LTHOT or need for NIMV, altered consciousness measured by GCS, and the use of accessory inspiratory muscles or paradoxical breathing upon ED arrival are tick variables. It means that by default they were stated as *No,* whereas selecting them with a tick changes their state to *Yes*. Age and baseline dyspnea (Grade 1-5) must be introduced in the integer format. P_CO2_ and pH are numerical values formatted with 1 and 2 decimal digits, respectively. The application does not allow data outside the established range or erroneous data entry, as stated on the help screen. If values for any of the variables included in the application are missing, the names of these variables as well as the estimated score and the risk category will appear in red. Moreover, it is indicated that the real value will be greater than or equal to the value on screen.

For instance, Figure 2 shows how data on a 55-year-old patient who arrives at ED with eCOPD, pH=7.35, P_CO2_=56.7, level of dyspnea-MRC=3, previous history of LTHOT or need for NIMV, use of accessory inspiratory muscles, and altered consciousness measured by GCS were introduced in the app running under Android. For a patient with these specific characteristics, the application estimates a value of 10 for the score that measures the risk of death during the first 7 days, which means a severe risk of death. The same patient, or another one with these characteristics, has an estimated value of 10 for the score that measures the risk of admission to ICU or IRCU, which is translated to a moderate risk of admission to ICU or IRCU.

Figure 3 shows how data on a patient arriving at ED with eCOPD who has a previous history of LTHOT or need for NIMV, use of accessory inspiratory muscles, altered consciousness measured by GCS, P_CO2_=46.7, level of dyspnea-MRC=4, and missing values for age and pH were introduced in the application running under Windows. For a patient with these specific characteristics, the application estimates a value greater than or equal to 10 for the score that measures the risk of death during the first 7 days, which means a severe or very severe risk of death. The same patient, or another one with these characteristics, has an estimated value of 8 or higher for the score that measures the risk of admission to ICU or IRCU, which is translated to a moderate or severe risk of admission to ICU or IRCU.

## Discussion

### Principal Findings

We have developed a computer application that implements the prediction models previously developed for 2 relevant adverse events in the short-term evolution of patients with eCOPD. The 2 adverse events selected as outcomes were mortality during hospital admission or within a week after the ED visit and admission to an ICU or IRCU during the eCOPD episode. The main strength of the app is that it is based on clinical predictive rules derived from models previously developed and validated for both outcomes.

The short-term evolution of patients with eCOPD is a critical issue regarding the health care provided at the EDs. Decision on medication, treatment, or hospitalization could be extremely benefited by any reliable information of the estimated risk of adverse evolution. Previous studies showed that relevant events in terms of bad evolution during the initial days would be death, ICU admission, need for IMV, cardiac arrest, need for NIMV if mechanical ventilation was not used at home before, or admission to an IRCU for some days [18,19]. Although some of the adverse events are obviously more severe than others, there is no continuum on the severity of all of them. Therefore, measuring the risk of any such events at the same time and through the same instrument could have a potential benefit over individual tools or crude predictive models.

As stated in the literature, the development and validation of prediction models require strict methodological norms [11]. When prediction models are developed, it may be necessary to make several assumptions regarding the structure of the data or the relation between covariates. If the aim is to apply the prediction model in practice, it is important to show that it is valuable when applied to new data, which is called validation. Internal validation evaluates the validity of the model when it is applied to data derived from the same sample in which they have been developed. Conversely, external validation examines the generalizability of the model to other samples. Usually, there are no data or funding available to do external validation. Hence, when a prediction model is developed, a good internal validation should be ensured at the least. The 2 logistic models we have selected to develop the app have been developed following proper procedures for derivation and validation, and they provide very good predictive validity. In addition, both models were derived from a large multicenter prospective cohort, and they use clinical data generally available in the ED and also at the primary care level.

Nowadays, the transference from clinical research to clinical practice is a relevant issue. The development of a clinical prediction rule goes one step further than predictive modeling. The development of a model does not mean that results predicted by the model would be used in daily clinical practice. Moreover, the success of a well-validated prediction model in practice will depend on 2 factors: its transfer to a reliable clinical rule and its availability in an easy-to-use tool. Implementation of a validated model into a user-friendly tool is a key step in developing risk models, which can increase the uptake of the model [20]. Thecalculator.co provides all kinds of free Web tools such as calculators, where one of the areas of interest is devoted to health [21]. Specifically for COPD, the website offers calculators for the well-known BODE Index (based on the body-mass index (B), the degree of airflow obstruction (O) and dyspnea (D), and exercise capacity (E), measured by the six-minute–walk test) [22] and for COPD stages classification by the Global Initiative for Chronic Obstructive Lung Disease GOLD guidelines [3]. Nevertheless, these tools are not all based on prediction models or clinical prediction rules.

Other studies have developed prediction models of evolution for patients with eCOPD [18,19] or have validated existing prediction models for other respiratory diseases [23,24]. Some of them have been translated into clinical prediction rules or scores for predicting short-term outcomes or stratifying patients based on their probability of adverse evolution [18,24,25]. However, as far as we know, none of them have been incorporated on an available and easy-to-use computer application that only needs to be downloaded to a computer device, such as a tablet or mobile phone, to be used. The implementation of a theoretical model into an easy-to-use application would allow its rapid and easy incorporation to the clinical management of eCOPD patients at the ED to guide their treatment. Nowadays, information systems are created differently across regions and countries. For the moment, we have stored our tool on a server so that it can be used in any health system in the world. As technology advances in each health system, our instrument could serve as the basis to automatically include information relative to the individual patient at the bed-side where decisions should be made. We are aware that until these tools are able to use information from electronic health record directly, emergency physicians will have to duplicate introduction of data, and this fact is a limitation for the generalization of the use of prediction models in clinical practice. We recommend lead efforts in this direction. Strictly, the use of these models in practice will allow us to properly validate them and, if necessary, update them.

Regarding other clinical fields, we have found some prediction rules that have been integrated into computer applications [26-28]. For instance, in the context of the Framingham Heart Study, several risk prediction models have been developed [29,30]. These risk scores are available either as an interactive calculator or a spreadsheet [26]. Another example is showed by Moreno-Cid et al, who performed a systematic review of the clinical prediction rules for the risk of Down syndrome based on ultrasound findings in pregnancy [31]. These authors showed that only 3 of the rules were validated (2 internally and 1 externally) and 4 of them were incorporated into a software application [32-35]. Moreover, a recent systematic review evaluated Web-based cardiovascular disease risk calculators in terms of clinical validity, understandability, and actionability [36]. The authors concluded that although the number of available Web-based tools is high, developers need to address actionability as well as clinical validity and understandability to improve usefulness. We believe that with regard to the prediction of evolution in the context of eCOPD, our software application verifies the 3 conditions highlighted by the authors, namely, validity, understandability, and actionability.

### Limitations and Future Work

This study inherits the limitations derived from the development of the 2 clinical prediction rules that have been translated into the application. These limitations were missing data for some key variables and the absence of biomarkers. These limitations were already previously cited and discussed in the original papers [9,10]. However, we would like to incorporate some discussion related to a third limitation, which was the lack of external validation of the developed predictive models. Authors of the clinical prediction rules for adverse events in the short-term evolution of patients with eCOPD asseverate that proper validation in future studies should further demonstrate their value in clinical practice. The use of the computer application that we present could easily allow for the storing of new data on patients attending to an ED with eCOPD, which could be posteriorly used to externally validate the original models and prediction rules in different populations. This easy-to-get bank of data would also allow for the description of types and profiles of patients attending an ED with an eCOPD. We should mention a new limitation, restricted to the app and not to the prediction rules, which is the fact that the selected outcomes were predefined. The application in its actual form does not allow for prediction of other outcomes apart from the ones included in the original prediction rules and clearly stated before. The prediction of any different outcome would require a previous development and validation of a new prediction rule and posterior incorporation into the app. Finally, we have reported the characteristics of the computer, the operating system, and the software versions under which the app has been developed and tested. We are not able to guarantee the correct performance of the application under different conditions.

### Conclusions

The proposed computer application shows how clinical prediction rules derived from multiple logistic regression models can be summarized into simple and easy-to-use tools that allow the estimation of the risk of short-term mortality and ICU or IRCU admission for patients with eCOPD. The app can be used in any computer device, including mobile phone or tablets, and it can guide the clinicians to a valid stratification of patients attending the ED with eCOPD.

## Abbreviations

COPD: chronic obstructive pulmonary disease
eCOPD: exacerbation of chronic obstructive pulmonary disease
ED: emergency department
GCS: Glasgow coma scale
ICU: intensive care unit
IMV: invasive mechanical ventilation
IRCU: intermediate respiratory care unit
IRYSS-COPD: Investigación en Resultados y Servicios de Salud–COPD
LTHOT: long-term home oxygen therapy
MRC: Medical Research Council
NIMV: noninvasive mechanical ventilation
P_CO2_: pressure of carbon dioxide
PrEveCOPD: prediction of evolution of patients with eCOPD
